# Associations between YKL-40 and markers of disease severity and death in patients with necrotizing soft-tissue infection

**DOI:** 10.1186/s12879-021-06760-x

**Published:** 2021-10-09

**Authors:** Morten Hedetoft, Marco Bo Hansen, Martin Bruun Madsen, Julia Sidenius Johansen, Ole Hyldegaard

**Affiliations:** 1 Department of Anaesthesia, Centre of Head and Orthopaedics, Copenhagen University Hospital, Rigshospitalet, Blegdamsvej 9, 2100 Copenhagen, Denmark; 2 Konduto ApS, Sani nudge, Copenhagen, Denmark; 3 Department of Intensive Care 4131, Copenhagen University Hospital, Rigshospitalet, Copenhagen, Denmark; 4 Department of Medicine, Herlev and Gentofte Hospital, Copenhagen University Hospital, Copenhagen, Denmark; 5 Department of Oncology, Herlev and Gentofte Hospital, Copenhagen University Hospital, Copenhagen, Denmark; 6 Department of Clinical Medicine, Faculty of Health and Medical Sciences, University of Copenhagen, Copenhagen, Denmark

**Keywords:** Chitinase-3-like-1 protein, Clinical endpoint, Fournier’s gangrene, Necrotizing fasciitis, Sepsis, Survival

## Abstract

**Background:**

Necrotizing soft-tissue infection (NSTI) is a severe and fast-progressing bacterial infection. Prognostic biomarkers may provide valuable information in treatment guidance and decision-making, but none have provided sufficient robustness to have a clinical impact. YKL-40 may reflect the ongoing pathological inflammatory processes more accurately than traditional biomarkers as it is secreted by the activated immune cells, but its prognostic yields in NSTI remains unknown. For this purpose, we investigated the association between plasma YKL-40 and 30-day mortality in patients with NSTI, and assessed its value as a marker of disease severity.

**Methods:**

We determined plasma YKL-40 levels in patients with NSTI (n = 161) and age-sex matched controls (n = 65) upon admission and at day 1, 2 and 3.

**Results:**

Baseline plasma YKL-40 was 1191 ng/mL in patients with NSTI compared with 40 ng/mL in controls (p < 0.001). YKL-40 was found to be significantly higher in patients with septic shock (1942 vs. 720 ng/mL, p < 0.001), and in patients receiving renal-replacement therapy (2382 vs. 1041 ng/mL, p < 0.001). YKL-40 correlated with Simplified Acute Physiology Score II (Rho 0.33, p < 0.001). Baseline YKL-40 above 1840 ng/mL was associated with increased risk of 30-day mortality in age-sex-comorbidity adjusted analysis (OR 3.77, 95% CI; 1.59–9.24, p = 0.003), but after further adjustment for Simplified Acute Physiology Score II no association was found between YKL-40 and early mortality.

**Conclusion:**

High plasma YKL-40 to be associated with disease severity, renal-replacement therapy and risk of death in patients with NSTI. However, YKL-40 is not an independent predictor of 30-day mortality.

**Supplementary Information:**

The online version contains supplementary material available at 10.1186/s12879-021-06760-x.

## Background

Necrotizing soft-tissue infection (NSTI) is a rare, severe and fast-progressing bacterial infection [1]. The incidence rate of NSTI is approximately two per 100,000 inhabitants/year in Denmark [2], but varies markedly across countries [3–6]. NSTI can be caused by a myriad of aerobic, anaerobic and facultative anaerobic bacteria [7], but predominantly by Group A Streptococcus in monomicrobial infections [8]. The clinical condition can rapidly progress into septic shock, multiple organ failure and death. Early recognition is key in NSTI, however timely diagnosis may be rendered as the initial symptoms such as swelling, pain and erythema are difficult to discriminate from less severe skin infections, consequently an initially misdiagnosis of 71% has been reported [9]. Despite early, radical and intensive multidisciplinary care the mortality remains high and largely unaltered in several years [2, 4].

Biomarkers may provide valuable information to the treating physician in treatment guidance and decision-making in NSTI patients, potentially improving morbidity and mortality. However, biomarkers have only been limited investigated in NSTI. No reliable diagnostic biomarkers exist [10, 11], hence surgical exploration persists the diagnostic golden standard although some imaging modalities may provide valuable diagnostic information [12–14]. Prognostic biomarkers have been sparsely assessed in patients with NSTI and none have provided sufficient robustness to have clinical impact [15–20]. Therefore, standard biochemistry and disease severity scores, such as Simplified Acute Physiology Score II (SAPS II), continues to provide the best level of prognostic risk-stratification and disease monitoring in the intensive care unit. Consequently, novel biomarkers are warranted in effort of increasing early diagnostic, therapeutic and prognostic risk-stratification in patients with NSTI.

YKL-40, also called chitinase-3-like-1 protein, may be an attractive prognostic biomarker in NSTI. YKL-40 is an acute phase protein secreted by various of immune cells, including macrophages, neutrophils and endothelial cells [21]. Of notice, proteomic analysis has indicated YKL-40 as a promising biomarker in patients with severe sepsis and septic shock [22]. Studies have suggested that high YKL-40 levels could be prognostic in patients with sepsis [23, 24]. However, the prognostic yields of YKL-40 remain unknown in NSTI. Extrapolation of YKL-40 results from patients with sepsis to NSTI may be questionable as the inflammatory response differ due to the combination of extensive tissue damage in NSTI and different pathogenesis in general. It would be of interests if high plasma YKL-40 levels could identify patients with NSTI with an increased risk of death as these patients might benefit from a more aggressive treatment protocol.

Therefore, we aimed to assess plasma YKL-40 as a prognostic biomarker in patients with NSTI and evaluate its accuracy as marker of disease severity and mortality. We hypothesized that high plasma levels of YKL-40 are associated with disease severity and 30-day mortality.

## Methods

### Study design and population

The present study was based on data of the Danish fraction from the prospective, observational INFECT study [8] (ClinicalTrials.gov; NCT01790698). We included patients with NSTI referred to Copenhagen University Hospital (Rigshospitalet, a tertiary hospital with a national NSTI service treatment), from February 2013 to October 2015.

We included adult patients (≥ 18 years) with a surgical confirmed NSTI diagnosis at either primary operation or at revision. We excluded patients in whom surgery did not reveal signs of NSTI. Control patients consisted of age-sex matched elective orthopedic patients without ongoing infection or inflammatory conditions (included between September 2014 and March 2015) [25]. The Strengthening the Reporting of Observational Studies in Epidemiology (STROBE) guidelines were followed in drafting of the present manuscript [26].

### Patient management

Our standardized multidisciplinary treatment protocol has in details been reported elsewhere [8]. In brief, the patients received frequent surgical debridement, initial broad-spectrum antibiotics (Clindamycin, Ciprofloxacin and Meropenem), intensive supportive care, hyperbaric oxygen treatment and immunoglobin G.

### Data collection

Predefined data were registered by dedicated personnel into an electronic database [27]. For the present study, clinical data included age, sex, comorbidities (cardiovascular disease, chronic kidney disease, chronic obstructive pulmonary disease, diabetes, immune deficiency, chronic liver disease, malignancy, peripheral vascular disease and rheumatoid disease), pre-existing condition (smoking, alcohol consumption, steroid treatment and immunosuppressing drugs), microorganism, standard biochemistry, severity scores (Simplified Acute Physiology Score II [SAPS II] and Sequential Organ Failure Assessment [SOFA]), ICU-length of stay, septic shock, amputation, renal-replacement therapy and vital status at day 30.

### YKL-40 measurement

Patient had blood samples collected into ethylenediaminetetraacetic acid (EDTA) sample tubes upon admission (baseline), and at the following three days (all between 8 a.m. and 12 a.m.). Samples were immediately put on ice and within 40 min centrifugated at 3500 rpm (2400G) for 10 min. The separated plasma was collected into cryo-tubes and stored at − 80 °C until analysis.

Plasma YKL-40 was quantified in duplicates by commercial enzyme-linked immunosorbent (ELISA) technique (Quidel, San Diego, CA, USA) at each of the four time points. The minimal detectable limit for YKL-40 was 10 ng/ml. The intra-assay CV was < 5% and the inter-assay CV was < 6% [28]. Plasma YKL-40 concentration was determined at Herlev Hospital without knowledge of the clinical data and prognosis of the patients.

### Outcomes

The primary outcome was association between baseline YKL-40 and 30-day mortality. Secondary analyses included the association between baseline YKL-40 and septic shock, renal-replacement therapy (RRT), amputation, SAPS II, SOFA score at day 1 and baseline blood lactate. We planned to run a sub-group analysis on YKL-40 levels according to infection with or without Group A Streptococcus.

### Statistical analysis

Test for normally were assessed with Shapiro–Wilks Test. Due to nonparametric distribution; continuous data are presented as medians (interquartile range, IQR) and categorial data as absolute numbers (percentage %). Continuous data were compared using Wilcoxon Rank Sum Test whereas categorical data were compared using Fisher’s Exact Test. We assessed correlations by Spearman’s rank correlation test. Receiver operating characteristic (ROC) curves were analyzed for baseline YKL-40 levels on 30-day mortality and area-under-curve (AUC) were reported. We categorized low versus high plasma YKL-40 level at admission according to both the median and the Youden Index optimal cutoff point [29]. Sensitivity, specificity, positive-predictive value (PPV) and negative-predictive value (NPV) were reported as prognostic test accuracy. Logistic regression analyses with Odds Ratio (OR) and 95% confidence intervals (95% CI) were used to assess the association between baseline YKL-40 and 30-day mortality. We adjusted for differences in age, sex, comorbidities (yes/no) and SAPS II score. Patients with missing SAPS II scores were excluded from logistic regression analyses. P-values are reported as exact unless < 0.001. P-values < 0.05 was considered statistically significant. Statistical analyses performed using RStudio Version 1.0.153 (RStudio, Inc.). Figures prepared in GraphPad Prism Version 8.0.2. (GraphPad Inc., La Jolla, CA, USA).

## Results

**Fig. 1:**
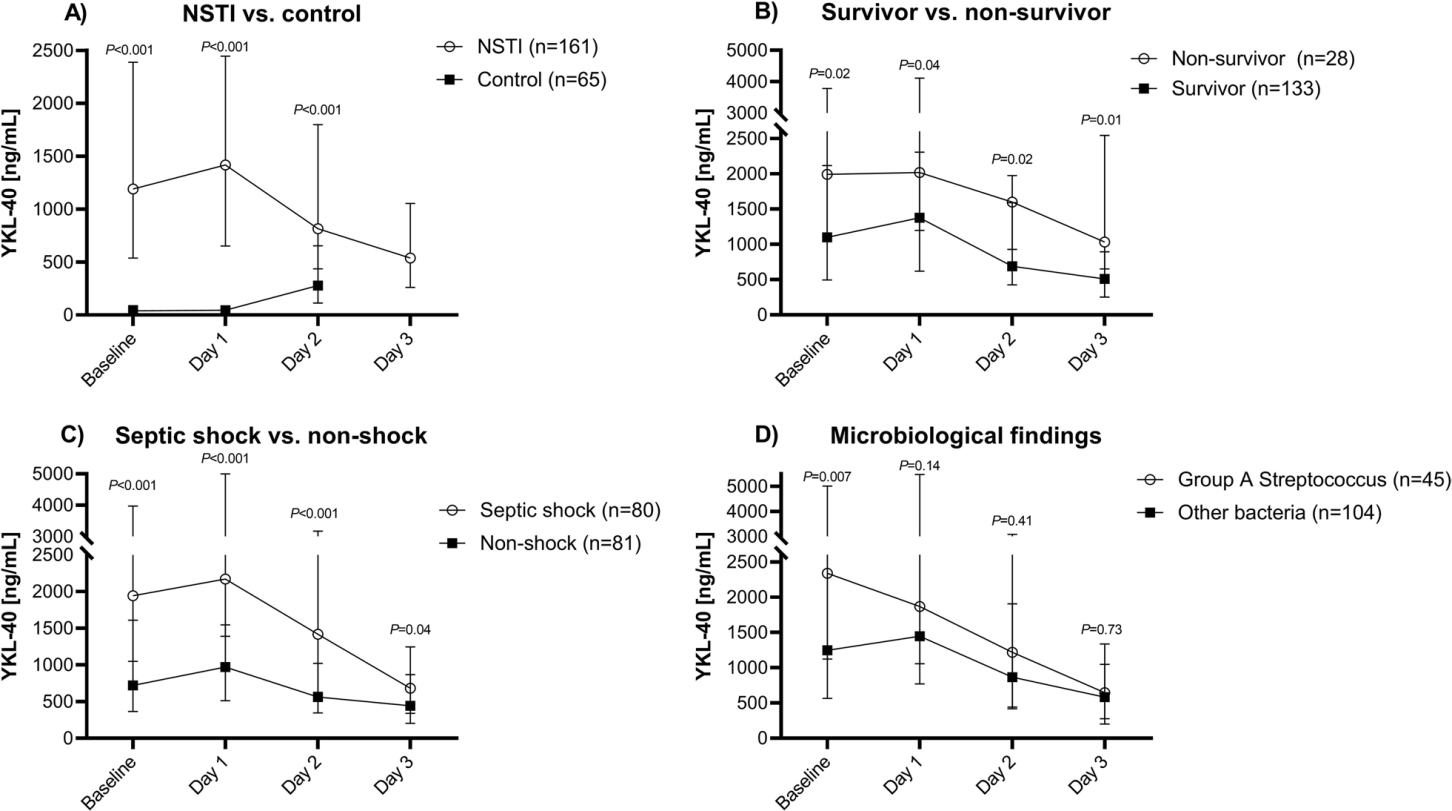
Plasma YKL-40 levels at admission (baseline), day 1, day 2 and day 3 in **a** NSTI vs. age- and sex-matched controls, **b** 30-day survivors vs. non-survivors, **c** septic shock vs. non-shock and **d** presence of Group A *Streptococcus* in blood and/or tissue. Note the two-segmented y-axis. Data are presented as medians with interquartile range. Comparisons performed by Wilcoxon Rank Sum Test. Septic shock was defined as s-lactate > 2 and use of vasopressor/inotropic agents. Microbiological findings were classified according to our protocol, in short, presumed pathogenic agent isolated at primary operation or immediately prior, up to three days after diagnosis. Group A streptococcus includes both monomicrobial GAS infections and polymicrobial infections including GAS. N = 12 patients had no positive microbiological findings

A total of 161 patients with NSTI (Additional file 1) and 65 age- and sex matched controls were included in the study. The clinical characteristics of the patients are presented in Table 1. No patients were lost to follow-up at day 30. Very high plasma YKL-40 levels were found at admission and at day 1, 2 and 3: 1191 ng/mL (IQR 538–2387), 1418 ng/mL (IQR 651–2445), 816 ng/mL (IQR 436–1798) and 537 ng/mL (IQR 261–1053), respectively. The controls had a baseline plasma YKL-40 of 40 ng/mL (IQR 29–87). Plasma YKL-40 was significantly higher in patients with NSTI compared to controls at admission, and at day 1 and 2 (Fig. 1A; controls had no YKL-40 determination at day 3).

**Table 1:** Characteristics of patients with necrotizing soft-tissue infection

	Entire cohort (n = 161)
*Demographics*	
Age, years	61 (53–69)
Sex, male	98 (61)
Body mass index, kg/m^2^	26 (24–31)
*Comorbidities*	
Cardiovascular disease	75 (47)
Chronic kidney disease	13 (8)
COPD	17 (11)
Diabetes	40 (25)
Immune deficiency	12 (7)
Chronic liver disease	9 (6)
Malignancy	14 (9)
Peripheral vascular disease	21 (13)
Rheumatoid disease	11 (7)
No comorbidities	49 (30)
*Pre-existing conditions*	
Active smoker^a^	49 (30)
High alcohol consumption^b^	23 (14)
Steroid treatment	21 (13)
Immunosuppressing drugs	15 (9)
*Biochemistry*	
Leukocyte count, 10^9^/L	16.2 (11.4–22.7)
C-reactive protein, mg/L	224 (153–309)
Procalcitonin, µg/L	8.6 (2.1–37.1)
Creatinine, µmol/L	121 (79–307)
Lactate, mmol/L	2.3 (1.3–4.7)
*Other*	
SOFA score^c^	8 (6–11)
SAPS II^d^	45 (35–54)
Septic shock^e^	80 (50)
ICU length of stay, days^f^	7 (4–13)
Amputation within 7 days^g^	27 (17)
RRT within 7 days	35 (22)
30-day mortality, n (%, 95% CI)	28 (17, 12–24)
90-day mortality, n (%, 95% CI)	37 (23, 17–30)

Continuous data are presented as medians (interquartile range, IQR) and categorial data as absolute numbers (percentage, %)

*COPD* chronic obstructive pulmonary disease, *ICU* intensive care unit, *RRT* renal replacement therapy

^a^Data were missing for 38 (24%) patients. ^b^High alcohol consumption defined as > 14 units of alcohol/week (female); > 21 units of alcohol/week (male), data were missing for 43 (27%) patients. ^c^Sequential Organ Failure Assessment (SOFA) Score (Day 1); data were missing for 3 (2%) patients. ^d^Simplified Acute Physiology Score II (SAPS II); data were missing for 4 (2%) patients. ^e^From the first 24 h after admission. Septic shock defined as lactate > 2 mmol/l and use of vasopressor or inotrope. ^f^Total ICU length of stay including patients subsequently transferred to ICUs in other hospitals. ^g^1 (50%) patients had NSTI on an extremity

### Association between YKL-40 and mortality

A total of 28 (17%) patients died within 30 days after admission. Among non-survivors, the median SAPS II score was 63 (51–77), and 18 patients had septic shock upon admission as compared to 41 (33–51) and 62, respectively, in survivors. In non-survivors, plasma YKL-40 at admission was significantly higher compared to survivors (1992 ng/mL (IQR 1107–3780) versus 1100 ng/mL (IQR 496–2117), p = 0.02). This finding persisted at day 1, 2 and 3 (Fig. 1B).

The optimal- and median cut-off levels of plasma YKL-40 at admission were 1840 ng/mL and 1191 ng/mL, respectively. In univariate analyses, plasma YKL-40 levels above the median and the optimal cut-off point were statistically associated with increased mortality (Table 2). These findings persisted in age, sex and comorbidity adjusted analyses, however when additionally adjusted for SAPS II no significant associations were observed (Table 2).

**Table 2:** Univariate and multivariate logistic regression analyses of 30-day mortality based on plasma YKL-40 concentration at admission according to the median and optimal cut-off values

	Plasma YKL-40 ng/mL	Unadjusted			Adjusted analysis: age, sex and comorbidity^a^			Adjusted analysis: sex, comorbidity and SAPS II^b^		
		OR	95% CI	*P*	OR	95% CI	*P*	OR	95% CI	*P*
Median	< 1191		1 (Reference)			1 (Reference)			1 (Reference)	
	≥ 1191	3.00	1.27–7.69	0.02	3.13	1.30–8.19	0.01	1.45	0.51–4.24	0.49
Optimal	< 1840		1 (Reference)			1 (Reference)			1 (Reference)	
	≥ 1840	3.55	1.55–8.48	0.003	3.77	1.59–9.24	0.003	1.75	0.62–4.89	0.29

*CI* confidence interval, *OR* odds ratio, *SAPS II* simplified acute physiology score II (data were missing for 4 (2%) patients, all survivors, these patients were excluded from the analyses)

^a^Comorbidity dichotomized (yes/no)

^b^Age included in SAPS II score

Plasma YKL-40 at admission showed a ROC-AUC of 0.64 (95% CI 0.52–0.76) on 30-day mortality (Additional file 2). This was lower than the SAPS II ROC-AUC of 0.86 (95% CI 0.80–0.93) and plasma lactate of 0.80 (95% CI 0.71–0.89). The prognostic accuracy of high YKL-40 is presented in Table 3.

**Table 3:** Left: Diagnostic accuracy of YKL40 (defined by Youden’s optimal cut-off point) in predicting 30-day mortality. Right: Spearman Rank Correlation between disease severity scores and baseline YKL-40

Accuracy		Correlation		
			Rho	*P*
Sensitivity	0.61 (95% CI 0.41–0.78)			
Specificity	0.70 (95% CI 0.61–0.77)	SAPS II	0.33	< 0.001
PPV	0.30 (95% CI 0.18–0.43)	SOFA Score	0.48	< 0.001
NPV	0.89 (95% CI 0.82–0.95)	Lactate	0.49	< 0.001
AUC-ROC	0.64 (95% CI 0.52–0.76)	Creatinine	0.46	< 0.001

Data are presented as fractions (95% Confidence Interval)

*AUC-ROC* area under the receiver operating characteristics curve, *CI* confidence interval, *NPV* negative predictive value, *PPV* positive predictive value, *SAPS II* simplified acute physiology score II, *SOFA Score* sequential organ failure assessment score day 1

### Association between YKL-40 and severity of disease

Plasma YKL-40 increased in most patients from admission to day 1, p < 0.001 (a median of 14 h (IQR 9–19) were observed from blood sampling at admission to day 1). Thereafter, plasma YKL-40 decreased from day 1 to day 2 and 3 (Fig. 1A). Admission plasma YKL-40 correlated with SAPS II and SOFA score, and with plasma lactate and creatinine (Table 3).

**Fig. 2:**
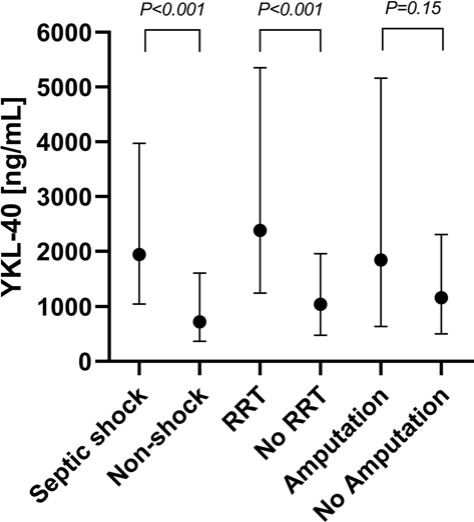
Plasma YKL-40 concentrations at admission in patients with necrotizing soft tissue infection according to patients with septic shock versus non-shock; RRT versus no RRT; and amputation versus non amputation (81 patients had NSTI located on an extremity). Data are presented as medians with interquartile range. Comparisons performed with Wilcoxon Rank Rum Test. *RRT* renal-replacement treatment

Plasma YKL-40 at admission was significantly higher in patients with septic shock (n = 80) (1942 ng/mL (IQR 1048–3970) versus 720 ng/mL (IQR 366–1610), p < 0.001) in non-shock patients (n = 81) (Fig. 1C). This finding persisted on day 1, 2 and 3 (Fig. 1C). Moreover, patients receiving RRT within 7 days had significantly higher YKL-40 compared to patient not requiring RRT (Fig. 2). Plasma YKL-40 at admission was significantly higher among patients infected with mono or polymicrobial Group A *Streptococcus* (n = 45) in blood and/or tissue (2338 ng/mL, IQR 1123–5007) compared to other types of NSTI (n = 104) (1246 ng/mL, IQR 565–2749), p = 0.007. However, this was not observed at day 1, 2 and 3 (Fig. 1D). No microbial findings were observed in 12 patients.

## Discussion

In this study, assessing the prognostic yields of plasma YKL-40 in patients with NSTI, we observed high plasma YKL-40 levels to be associated with increased risk of death and severity of the disease. However, the association was not statistically significant when adjusted for SAPS II. We found the highest plasma YKL-40 levels at admission among patients in septic shock, patients requiring RRT and patients infected with Group A *Streptococcus*.

We compared the prognostic accuracy of 30-day mortality of plasma YKL-40 with plasma lactate, known to be associated with increased mortality [8, 15], and with SAPS II which is among the most common disease severity score used in the intensive care unit. We found a lower ROC-AUC for YKL-40 compared to both plasma lactate and SAPS II, indicating that the prognostic information of plasma YKL-40 is not be better than the existing evaluation tools. Yet, caution is required when exclusively comparing AUC between prognostic indicators, as AUC reflects the overall performance of all possible cut-off values including those that will never be clinically applied. Therefore, we addressed the prognostic accuracy as sensitivity, specificity, PPV and NPV according to the optimal cut-off point. Here we observed a low sensitivity but moderate good specificity and NPV indicating patients with plasma YKL-40 levels below 1840 ng/mL to have an 89% chance of surviving the first 30 days. We observed a reduction in YKL-40 over time potentially reflecting a generally improved clinical condition among patients, but such result must be cautiously interpreted as a risk of dilution occurs when measuring across time, as the most critically ill patients who have highest YKL-40 levels at admission, are probably also the patients who first decease.

To our knowledge, no prognostic biomarkers have yet provided enough robustness to provide a clinical impact in NSTI treatment. Pentraxin-3, a molecule released by various immune cells at the onset of inflammation, has earlier demonstrated a ROC-AUC of 0.66 in predicting 180-day mortality with a sensitivity of 0.69 and a specificity of 0.56 in patients with NSTI [18]. Additionally, the prognostic accuracy of suPAR in predicting 90-day mortality has shown a ROC-AUC of 0.77, but the combination of suPAR and SAPS II was not superior compared to SAPS II alone (ROC-AUC 0.87) [17]. The burden of disease, the unaltered mortality rates and the lack of robust biomarkers all highlight the need for studies evaluating novel biomarkers potentially capable of reducing disease morbidity and mortality.

We included age, sex, comorbidity and disease severity as covariates in logistic regression analyses, as these are among the most documented prognostic risk factors of not surviving NSTI [2, 4, 30–36]. Other covariates such as septic shock [34, 37] need of inotropes and RRT [35, 38] could have been included as covariates, but according to the ‘one-in-ten rule’ on the number of covariates in logistic analyses, only three covariates entered multivariate analyses [39, 40]. Furthermore, these are included in the SAPS II. We observed high plasma YKL-40 at admission to be associated with increased mortality in univariate and age-sex adjusted analyses, but not after adjusting for SAPS II indicating that YKL-40 is not an independent predictor of mortality. Yet, plasma YKL-40 could provide an early prognostic information for the treating physician as SAPS II needs 24 h of admission in the intensive care unit before a complete score can be calculated, and some variables may be missing rendering the calculation. Although no quick/bed-side YKL-40 assays exist, well-performing and rapid multiplex assays (≈75 min) have been developed [41].

Severe infection such as patients with septic shock and patients requiring RRT are associated with mortality [34, 37, 38]. Therefore, we compared differences in plasma YKL-40 according to presence of septic shock or requiring RRT. We found patients with septic shock to have higher plasma YKL-40 levels at admission and the following three days compared to the levels in non-shock patients. Similar patterns were observed in patients requiring RRT and patients not receiving RRT. These results may reflect a more pronounced immune cell activity and a higher burden of disease in these patients. Additionally, patients with Group A *Streptococcus* were found to have significantly higher plasma YKL-40 levels at admission compared to patients with other types of NSTI, potentially reflecting a higher rate of septic shock in these individuals as earlier demonstrated [8]. These observations suggest that burden of disease is increased in patients with high plasma YKL-40 and is underlined by the positive correlations between baseline YKL-40 and SAPS II, SOFA score and plasma lactate in the present study.

In healthy individuals, plasma YKL-40 is approximately 40 ng/mL (2.5–97.5% CI 14–155) [28] which is comparable to our observation of 40 ng/mL (IQR 29–87). We demonstrated approximately 30 times higher plasma levels in patients with NSTI compared to controls, indicating that severe bacterial infections influence YKL-40 secretion from inflammatory cells. Others have earlier found plasma YKL-40 levels in patients with sepsis and septic shock to be approximately 700–1000 and 1200–2200 ng/mL, respectively [23]. These results are in accordance with the present plasma YKL-40 levels of 720 ng/mL in non-shock NSTI patients and 1942 ng/mL in NSTI patients with septic shock. Additionally, in a cohort of 89 patients with *Streptococcus pneumoniae* bacteremia the median YKL-40 was 342 ng/mL and was associated with in-hospital mortality [42], whereas in 289 hospitalized patients with community-acquired pneumonia, plasma YKL-40 was higher in patients with severe infection and associated with short- and long-term mortality [43]. Interestingly, a study including both experimental and human data has revealed that YKL-40 may be able to discriminate been sepsis-induced acute kidney injury and sepsis without acute kidney injury [44]. In that context, patients with Puumala hantavirus infection—a virus infection typically inducing acute kidney injury—YKL-40 was more pronounced during the acute phase, and correlated with the degree of inflammation and severity of acute kidney injury [45]. These finding are in agreement with the present study demonstrating significantly higher levels plasma YKL-40 in those receiving RRT within 7 days from admission.

Furthermore, plasma YKL-40 has recently been demonstrated to be a biomarker of future infectious diseases in the general population with adjusted hazard ratio of future episodes of skin infections and sepsis of 1.76 and 1.90, respectively [46]. These findings emphasize plasma YKL-40 as a promising biomarker for further research in infectious diseases including NSTI.

YKL-40 is not a sole biomarker for infectious disease [21]. Plasma YKL-40 has been studied in patients with different types of cancer and high levels are associated with short overall survival [47]. Furthermore, high plasma YKL-40 levels are also associated with poor prognosis in non-cancerous diseases including cardiovascular, respiratory, digestive, endocrine, nervous, urinary and skeletal diseases [21]. In patients with infectious disease plasma YKL-40 may reflect the ongoing pathological inflammatory processes more accurately than traditionally biomarkers such as CRP (produced by hepatocytes), since YKL-40 is secreted by activated immune cells including macrophages, neutrophils and endothelial cells [21]. YKL-40 is considered an acute-phase protein and is stimulated by INF-γ and IL-6 [48, 49]. We have earlier demonstrated plasma IL-6 to be associated with NSTI disease severity and particular elevated in patients with septic shock and β-hemolytic streptococcal infection, potentially indicating a relationship between YKL-40/IL6 and burden of disease in NSTI [20]. Despite some regulators of YKL-40 have been defined during the last decades [21], a great knowledge gap exists on its pathophysiological role and functional effects in infectious diseases including sepsis and NSTI [50, 51]. Future research may include evaluation of YKL-40 in non-necrotic soft-tissue lesions such as erysipelas, cellulitis or cutaneous abscesses in effort of investigating the diagnostic yields of YKL-40.

To our knowledge this is the first study to report plasma YKL-40 levels in patients with NSTI. A strength of the present study is the sample size and that no patients were lost to follow-up. We had broad inclusion criteria and only few exclusion criteria. Additionally, the clinical data was prospectively collected by dedicated personnel using a predefined and standardized protocol [27]. Lastly, the laboratory conducting the plasma YKL-40 analyses was blinded for patient type and outcome. However, some limitations exist: First, since this is an observational study there is a potential risk of missing unknown confounders. Second, although our study center has a national NSTI service treatment and thereby increasing the heterogeneity, we may be missing the patients who are too ill for transportation consequently introducing an important selection bias into our analyses. Third, several patients were either on immunosuppressing drugs or steroid treatment at admission theoretically impacting YKL-40 concentration. Last, no predefined protocol regarding biomarker analyses was published.

## Conclusions

We found that plasma YKL-40 levels were associated with disease severity and risk of death in patients with NSTI. However, YKL-40 is not an independent predictor for 30-day mortality.

## Supplementary Information

**Additional file 1.** Flow chart of patients included in the study. Patients with suspected NSTI were screened for eligibility. Patients were excluded if they did not meet the criteria of inclusion. After inclusion, patients’ files were reviewed and 7 were deemed non-NSTI due to no intraoperative signs of necrotizing soft tissue infection. 2 patients did not have blood samples available for analyses. 1 patient was discontinued as informed consent was not obtainable.

**Additional file 2.** Receiver operating characteristics curve of 30-day mortality in patients with necrotizing soft-tissue infection according to plasma YKL-40 and lactate at admission and SAPS II. SAPS II; Simplified Acute Physiology Score II.

## Data Availability

The datasets analysed during the current study available from the corresponding author on reasonable request.

## Abbreviations

AUC: Area-under-curve
CI: Confidence interval
ELISA: Enzyme-linked immunosorbent assay
IQR: Interquartile range
NSTI: Necrotizing soft-tissue infection
NPV: Negative-predictive value
OR: Odds ratio
PPV: Positive-predictive value
ROC: Receiver operating characteristic
RRT: Renal-replacement therapy
SAPS II: Simplified acute physiology score II
SOFA: Sequential organ failure assessment
