# Supplementary material for: Associations between YKL-40 and markers of disease severity and death in patients with necrotizing soft-tissue infection
Source: BMC Infect Dis. 2021 Oct 9;21:1046. doi: 10.1186/s12879-021-06760-x (PMC8502346; doi:10.1186/s12879-021-06760-x)
Supplement: Supplementary file 1 — Additional file 1. Flow chart of patients included in the study. Patients with suspected NSTI were screened for eligibility. Patients were excluded if they did not meet the criteria of inclusion. After inclusion, patients’ files were reviewed and 7 were deemed non-NSTI due to no intraoperative signs of necrotizing soft tissue infection. 2 patients did not have blood samples available for analyses. 1 patient was discontinued as informed consent was not obtainable. [file 12879_2021_6760_MOESM1_ESM.docx]

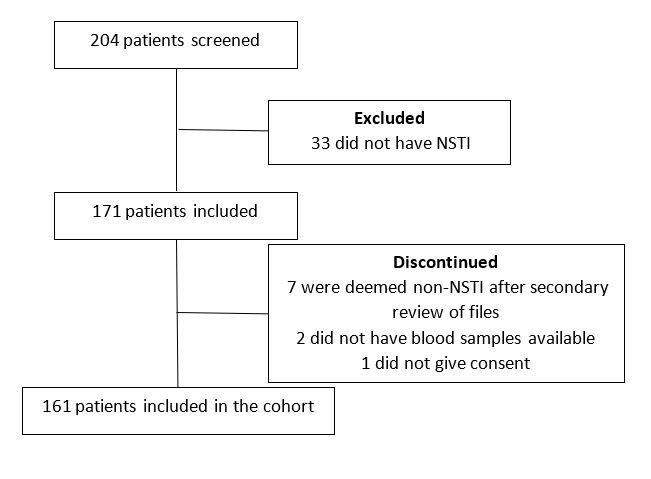


**Additional File 1.** Flow chart of patients included in the study. Patients with suspected NSTI were screened for eligibility. Patients were excluded if they did not meet the criteria of inclusion.
After inclusion, patients’ files were reviewed and 7 were deemed non-NSTI due to no intraoperative signs of necrotizing soft tissue infection. 2 patients did not have blood samples available for analyses. 1 patient was discontinued as informed consent was not obtainable.
