# Supplementary material for: Associations between YKL-40 and markers of disease severity and death in patients with necrotizing soft-tissue infection
Source: BMC Infect Dis. 2021 Oct 9;21:1046. doi: 10.1186/s12879-021-06760-x (PMC8502346; doi:10.1186/s12879-021-06760-x)
Supplement: Supplementary file 2 — Additional file 2. Receiver operating characteristics curve of 30-day mortality in patients with necrotizing soft-tissue infection according to plasma YKL-40 and lactate at admission and SAPS II. SAPS II; Simplified Acute Physiology Score II. [file 12879_2021_6760_MOESM2_ESM.docx]

**
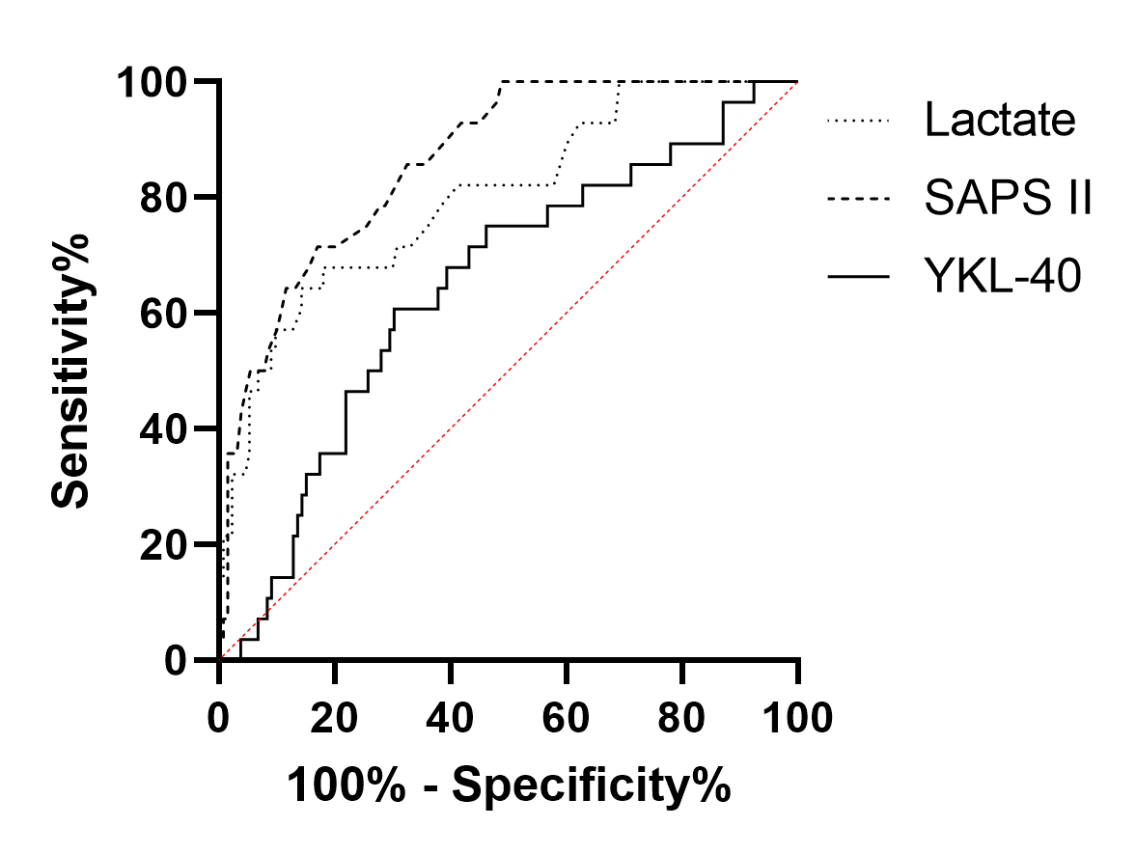
**

**Additional File 2.** Receiver operating characteristics curve of 30-day mortality in patients with necrotizing soft-tissue infection according to plasma YKL-40 and lactate at admission and SAPS II. SAPS II; Simplified Acute Physiology Score II.
